# Immune activity and biodistribution of polypeptide K237 and folic acid conjugated amphiphilic PEG-PLGA copolymer nanoparticles radiolabeled with ^99m^Tc

**DOI:** 10.18632/oncotarget.12850

**Published:** 2016-10-24

**Authors:** Zelai He, Xiangyu Zhang, Jingwen Huang, Yufeng Wu, Xuanzhang Huang, Jie Chen, Junyong Xia, Hao Jiang, Jing Ma, Jian Wu

**Affiliations:** ^1^ The Second Affiliated Hospital and Yuying Children's Hospital of Wenzhou Medical University, Wenzhou, China; ^2^ Ultrasonic Department, Shanghai Songjiang Center Hospital, Shanghai, China; ^3^ The First Affiliated Hospital of Bengbu Medical College, Bengbu, China; ^4^ Department of Pathology, Jining No.1 Peoples' Hospital, Jining, China; ^5^ Department of Internal Medicine, Affiliated Cancer Hospital of Zhengzhou University, Henan Cancer Hospital, Zhengzhou, China; ^6^ Department of Nuclear Medicine, The Affiliated Provincial Hospital of Anhui Medical University, Hefei, China

**Keywords:** K237, folate, PEG-PLGA, nanoparticles, biodistribution

## Abstract

In a previous study, amphiphilic copolymer, polypeptide K237 (HTMYYHHYQHHL) and folic acid (FA) modified poly(ethylene glycol)-poly(lactic-co-glycolic acid) (K237/FA-PEG-PLGA) nanoparticles were developed and studied as a drug carrier. To further promote the clinical application of K237/FA-PEG-PLGA nanoparticles and provide guidance for future research, we need to examine their specific biodistribution *in vivo*. In this study, K237/FA-PEG-PLGA nanoparticles were effectively labeled by a direct method with Technetium-99m (^99m^Tc) using stannous chloride as a reducing agent. The optimal stability of the labeled nanoparticles was determined by evaluating their radiochemical purity in serum, physiological saline, diethylenetriaminepentaacetic acid (DTPA) and cysteine solutions. The affinity of ligands and receptors was elicited by cell binding and blocking experiments in KDR/folate receptor high expressing SKOV-3 ovarian cancer cells. The nanoparticles biodistribution was studied after intravenous administration in healthy mice xenografted with SKOV-3 cells. A higher percent injected dose per gram of tissue (% ID/g) was observed in liver, kidney, spleen, blood and tumor at 3 and 9 h post-injection. Scintigraphic images revealed that the radioactivity was mainly concentrated in tumor, liver, kidney and bladder; and in the heart, lung, and muscle was significantly lower at 3 h. The radioactivity distribution in the images is consistent with the *in vivo* biodistribution data. Our works demonstrated that K237/FA-PEG-PLGA nanoparticles have great potential as biodegradable drug carriers, especially for tumors expressing the folate and KDr receptor.

## INTRODUCTION

Technetium-99m (^99m^Tc; half-life, 6.02 h; γ-energy, 140.5 KeV) is a commonly used nuclide in biodistribution, drug tracing and molecular imaging applications due to its low cost, excellent availability, little impact on drug biochemical properties, photon energy that is nearly ideal for single-photon emission computed tomography (SPECT) and low absorbed-dose burden to the patients.

In this study, an amphiphilic polymer, namely peptides K237 (HTMYYHHYQHHL) (target the vascular endothelial growth factor receptor-2, abbreviation: VEGFR-2 or KDR) and folic acid (FA) modified poly(ethylene glycol)-poly(lactic-co-glycolic acid) (K237/FA-PEG-PLGA), was used to prepare nanoparticles (NPs) which can serve as favorable carriers for the delivery of certain drugs [1–4]. In this delivery system, the covalent conjugation of K237 and FA to the NPs confers them the ability to actively bind the KDR receptor and folate receptor (FR). Binding to the receptors leads to a greater uptake of the NPs by KDR/FR-overexpressing tumor cells while virtually ignoring normal tissues, thereby, increasing tumor cell specificity, greatly enhancing antitumor efficacy, and dramatically reducing potentially dangerous side effects [5–8]. Polyethylene glycol (PEG) functions as the outer corona and prolongs the circulation time of the NPs in blood by reducing non-specific interactions with blood components [9–17]. The PLGA hydrophobic component serves as a reservoir for lipophilic drug, while the anionic component provides the ability to strong electrostatic interaction with cationic drugs [18, 19]. However, the biodistribution of K237/FA-PEG-PLGA NPs has not yet been specifically and quantized studied in ovarian cancer model by using radioisotope ^99m^Tc. Accordingly, we used the radioisotope ^99m^Tc to label the delivery system and study the distribution of radioactivity in various tissues following administration of the labelled NPs by performing gamma imaging of the whole body at predetermined time point.

## RESULTS AND DISCUSSION

### Physics characterization of NPs

**Figure 1:**
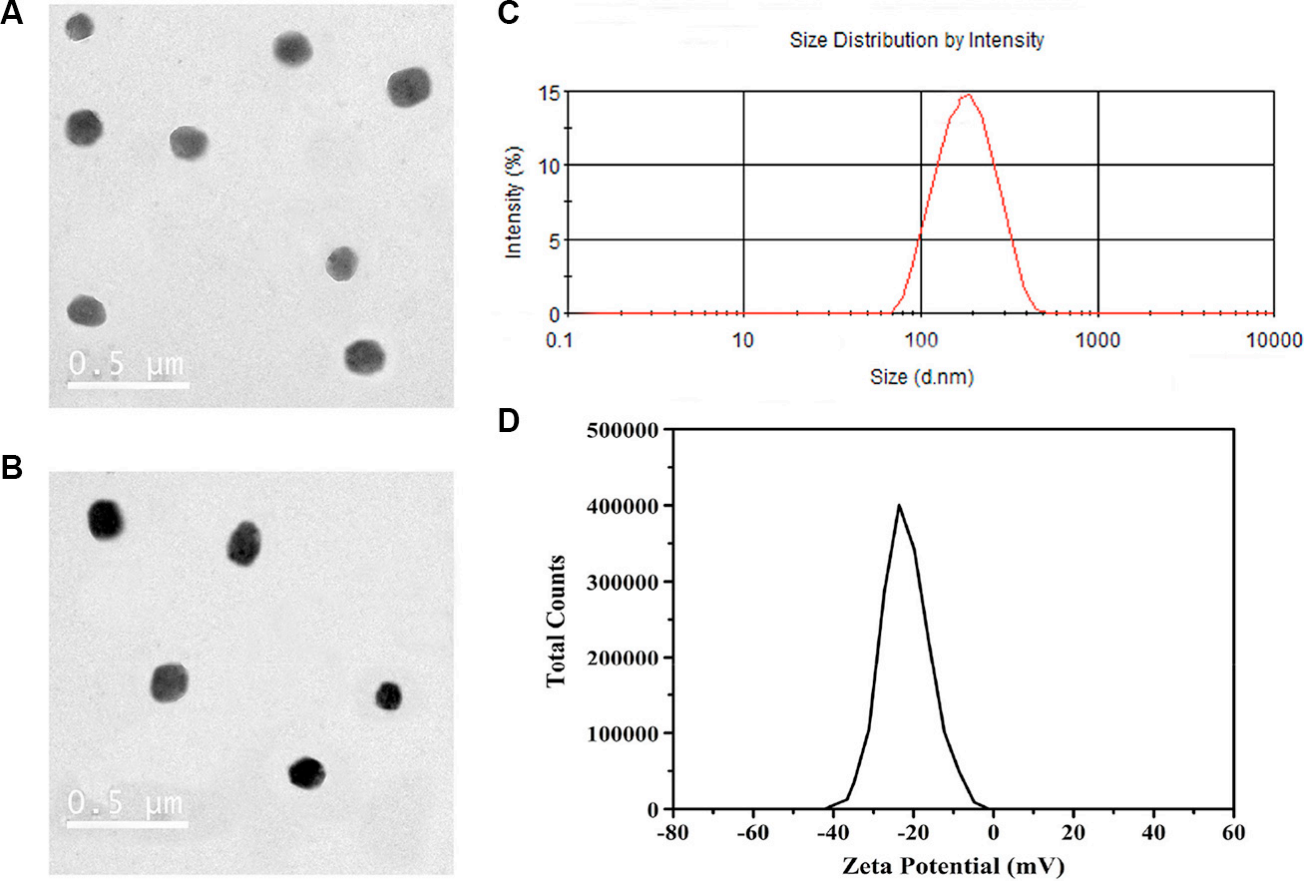
TEM and DLS characterization of the NPs The K237/FA-PEG-PLGA (LA/GA = 60/40) NPs (**A**) and ^99m^Tc-K237/FA-PEG-PLGA (LA/GA = 60/40) NPs (**B**) are spherical and uniform size. K237/FA-PEG-PLGA (LA/GA = 60/40) NPs have a diameter of about 130 nm (**C**), and negative zeta potential (**D**).

The K237/FA-PEG-PLGA NPs have a rigid structure due to ionic interaction among the base amino groups at the amino-terminus of PEG-PLGA. The mean diameter of the K237/FA-PEG-PLGA (LA/GA = 60/40, 70/30, 80/20) NPs was 128.7 ± 13.9, 114.6 ± 10.3, and 104.2 ± 9.6 nm, respectively, and the ζ potential was −23.53 ± 2.79, −18.71 ± 1.84, and −16.91 ± 2.08 mV, respectively. Meantime, the polydispersity was 0.18 ± 0.03, 0.17 ± 0.03 and 0.14 ± 0.02. respectively. The mean size and polydispersity index values indicate the narrow size and homogenous distributions of the particles. The ζ potential values indicate the stability of the nanodispersions. The presence of PEG chains in the outer shell layer was used to improve the stability and biocompatibility of the NPs [20–23]. A TEM photograph, presented in Figure 1, shows that the NPs were approximately round, smooth, uniform size and exhibited no agglomeration. And there were not obvious changes after ^99m^Tc label NPs.

### Radiolabeling of NPs with ^99m^Tc

The NPs were labeled with ^99m^Tc with a high labeling efficiency by a direct method. In the labeling process, the amount of stannous chloride and pH value were critical factors influencing labeling efficiency. The amount of stannous chloride affected the ratio of reduced/hydrolyzed (R/H) ^99m^Tc and free ^99m^Tc. Thus, higher amounts of stannous chloride could lead to formation of radioactive colloids which are undesirable, while lower amounts of stannous chloride could lead to poor labeling efficiency. The influence of stannous chloride on the labeling efficiency and the R/H of ^99m^Tc is shown in Table 1. In this study, the aim was to determine the optimal amount of stannous chloride required for higher labeling efficiency with lower free ^99m^Tc and R/H of ^99m^Tc. The study found that 20 μg was the optimal amount required for all NPs preparations. The optimal pH value and incubation time of the NPs, which can obtain high labeling efficiency, was 7.0 and 15 min, respectively. To optimize the above parameters, quality control tests were performed by TLC using ITLC strips.

**Table 1:** Influence of the amount of stannous chloride on the labeling efficiency of NPs

SnCl_2_·2H_2_O (μg)	20	30	50	100
K237/FA-PEG-PLGA (LA/GA = 60/40)					
% Labeled (mean ± SD)	97.95 ± 1.13	95.88 ± 2.73	94.3 ± 1.90	86.92 ± 2.85
% colloids (mean ± SD)	0.37 ± 0.35	1.16 ± 0.87	1.77 ± 0.55	2.71 ± 1.83
% free (mean ± SD)	1.68 ± 0.44	2.96 ± 1.07	3.93 ± 1.61	10.37 ± 3.32
K237/FA-PEG-PLGA (LA/GA = 70/30)					
% Labeled (mean ± SD)	96.56 ± 3.66	94.12 ± 3.12	93.58 ± 3.16	84.12 ± 3.24
% colloids (mean ± SD)	1.08 ± 0.36	2.03 ± 0.82	2.37 ± 1.00	4.06 ± 2.75
% free (mean ± SD)	2.36 ± 1.42	3.75 ± 1.28	4.05 ± 0.78	11.82 ± 2.47
K237/FA-PEG-PLGA (LA/GA = 80/20)					
% Labeled (mean ± SD)	95.40 ± 2.29	94.39 ± 1.93	93.26 ± 3.19	82.98 ± 2.70
% colloids (mean ± SD)	1.73 ± 0.72	2.099 ± 0.54	2.56 ± 0.82	4.75 ± 2.50
% free (mean ± SD)	2.87 ± 0.72	3.52 ± 1.86	4.18 ± 2.04	12.27 ± 3.43

### Stability of labeled NPs *in vitro*

The ^99m^Tc-labeled NPs were assessed for their stability *in vitro* after incubation with healthy human serum and 0.9% NaCl solutions. These conditions were selected to mimic the *in vivo* internal environment, physiological pH, *in vitro* storage and applied environment. The human serum contains many kinds of proteins which can chelate and bind to ^99m^Tc, affect the stability of the labeled NPs in blood when the labeled NPs are injected intravenously. If the labeled NPs were stable in serum, markers can be used for *in vivo* biodistribution studies.

**Figure 2:**
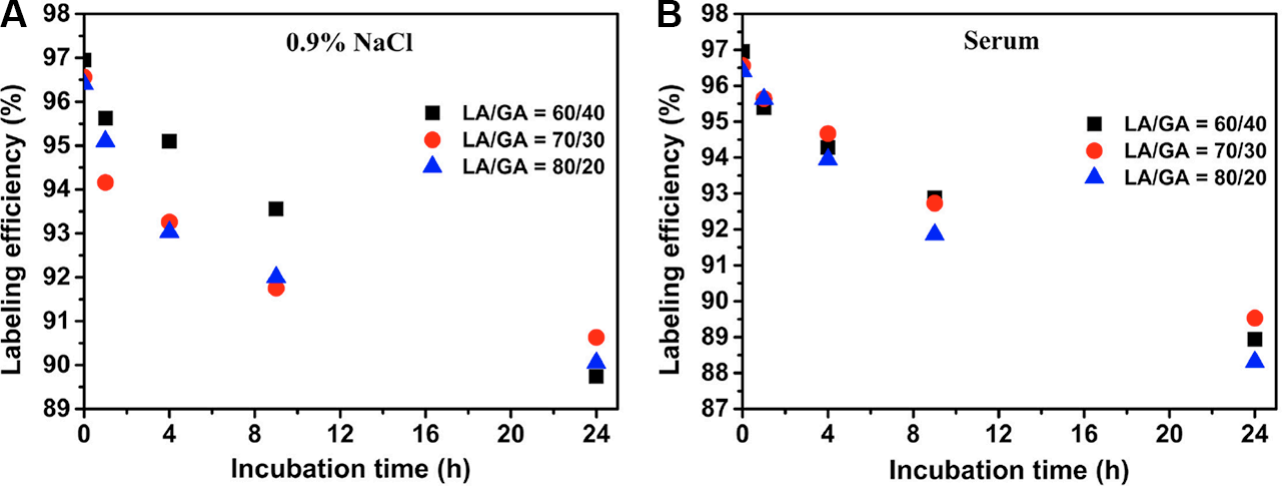
*In vitro* stability studies of the ^99m^Tc-K237/FA-PEG-PLGA NPs in (A) physiological saline and (B) human serum

The labeling efficiency of the three kinds of NPs, namely LA/GA = 60/40, 70/30 and 80/20, in a 0.9% NaCl solution was 95.1%, 94.26%, 93.03%, respectively, at 4 h, and 89.74%, 90.63%, 90.05%, respectively, at 24 h (Figure 2A). The labeling efficiency in serum was 94.28%, 94.67%, 93.95%, respectively, at 4 h, and 88.94%, 89.53%, 88.31%, respectively, at 24 h (Figure 2B). These results indicate that the three kinds of NPs were quite stable in serum and normal saline within 24 h.

### DTPA and cysteine challenge

**Figure 3:**
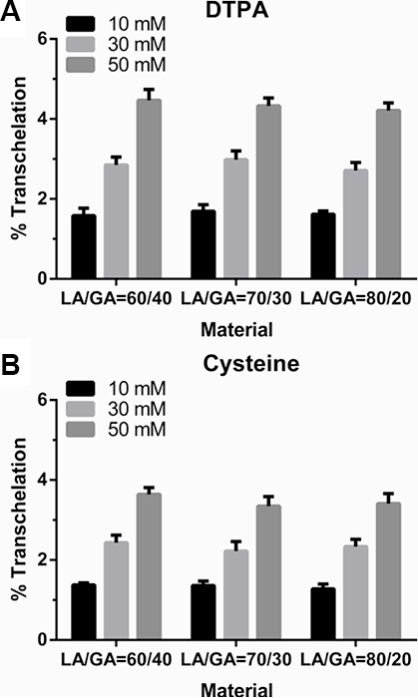
Determination of the *in vitro* stability of the ^99m^Tc-K237/FA-PEG-PLGA (LA/GA = 60/40, 70/30, 80/20) NPs by the (A) DTPA and (B) cysteine challenge test

The strength of the ^99m^Tc binding to NPs in ^99m^Tc-labeled NPs was tested with 10, 30, 50 mM of DTPA or cysteine, which may indirectly confirm that no trans-chelation of the metal occurred in such physiological environment. The results indicated that the labeling efficiency of the NPs was not altered much in presence of DTPA (Figure 3A) or cysteine (Figure 3B). Indeed, an evaluation of these results revealed that only 1%–2% transchelation occurred when incubated with 10 mM of DTPA or cysteine; while incubation for 1 h with 50 mM of DTPA or cysteine, the transchelation was only around 4–5% with DTPA and less than 4% with cysteine, indicating the high stability of the labeled NPs.

### Cell binding and blocking experiments *in vitro*

The binding specificity of the ^99m^Tc-K237/FA-PEG-PLGA NPs to the SKOV-3 cell line is shown in Tables 2–4. The results of the binding specificity tests indicated that the binding of the ^99m^Tc-labeled NPs to FR-expressing cells was receptor-mediated, because the receptors could be blocked and their saturation by pre-incubation with an excess of FA can significantly decreased the binding of the radiolabeled NPs. However, the amount of the free ^99m^TcO_4_^−^ binding to cells was negligible. The cell-associated immune activity of the ^99m^Tc-K237/FA-PEG-PLGA (LA/GA = 60/40, 70/30, 80/20) NPs binding to the SKOV-3 cell line was 31.75%, 32.79% and 34.01%, respectively. The highest specific binding rate of the ^99m^Tc-K237/FA-PEG-PLGA (LA/GA = 60/40, 70/30, 80/20) NPs binding to SKOV-3 cells was 32.28%, 34.13%, 36.72%, respectively. Additionally, the total and specific binding rates declined with a decrease of the density of the SKOV-3 cells. These results indicate that the labeled NPs have good bioactivity and specificity. Specifically, the ^99m^Tc-K237/FA-PEG-PLGA (LA/GA = 80/20) NPs have the best bioactivation and specificify of the three kinds of NPs evaluated, although the difference was not statistically significant.

**Table 2:** The ^99m^Tc-K237/FA-PEG-PLGA (LA/GA = 60/40) NPs binding rate (%) of SKOV-3 cells

Number of SKOV-3 cells	^99m^TcO4^–^	LA/GA = 60/40		
		Total binding	Non-specific binding	Specific binding
5 × 10^6^	2.59 ± 0.47	36.14 ± 1.0	3.86 ± 0.13	32.28
1 × 10^6^	2.30 ± 0.21	33.41 ± 0.18	3.57 ± 0.1	29.84
1 × 10^5^	1.89 ± 0.53	30.48 ± 1.10	2.52 ± 0.32	27.96
5 × 10^4^	1.58 ± 0.55	25.89 ± 1.70	1.69 ± 0.06	24.2
1 × 10^4^	1.62 ± 0.71	21.37 ± 1.50	1.74 ± 0.88	19.63

**Table 3:** The ^99m^Tc-K237/FA-PEG-PLGA (LA/GA = 70/30) NPs binding rate (%) of SKOV-3 cells

Number of SKOV-3 cells	LA/GA = 70/30		
	Total binding	Non-specific binding	Specific binding
5 × 10^6^	37.89 ± 0.67	3.76 ± 0.21	34.13
1 × 10^6^	35.99 ± 2.09	3.69 ± 0.20	32.3
1 × 10^5^	29.65 ± 1.46	2.93 ± 0.16	26.72
5 × 10^4^	24.73 ± 0.50	2.09 ± 0.03	22.64
1 × 10^4^	20.75 ± 1.91	1.71 ± 0.25	19.04

**Table 4:** The ^99m^Tc-K237/FA-PEG-PLGA (LA/GA = 80/20) NPs binding rate (%) of SKOV-3 cells

Number of SKOV-3 cells	LA/GA = 80/20		
	Total binding	Non-specific binding	Specific binding
5 × 10^6^	40.56 ± 2.31	3.84 ± 0.47	36.72
5 × 10^5^	33.08 ± 0.57	3.34 ± 0.40	29.74
1 × 10^5^	29.95 ± 1.52	2.83 ± 0.02	27.12
5 × 10^4^	27.17 ± 1.08	2.26 ± 0.06	24.91
1 × 10^4^	21.85 ± 0.82	1.92 ± 0.79	19.93

### *In vivo* studies

### Biodistribution studies

Data on the comparative biodistribution of the ^99m^Tc-labeled NPs in male BALB/c nu/nu mice xenografted with SKOV-3 cells at 3, and 9 h are presented in Table 5. The average radioactivity in the liver, and spleen, was 23.1 and 13.28%, respectively, at 3 h, and 6.50 and 2.45%, respectively, at 9 h. The ^99m^Tc- K237/FA-PEG-PLGA (LA/GA = 80/20) NPs were found to be mainly concentrated in the mononuclear phagocyte system (MPS). The low level of radioactivity detected in the stomach suggests that the release of ^99m^Tc *in vivo* was negligible. The high radioactivity in kidney, indicates that the ^99m^Tc-labeled NPs were mainly metabolized through the urinary system. Meanwhile, the high radioactivity detected in blood, reveals the long cycle effects of the NPs, and suggests that it may be better to target tumors by the enhanced permeability and retention effect (EPR) and the binding of ligand and receptor.

**Table 5:** Biodistribution of the ^99m^Tc-labeled NPs in male BALB/c nu/nu mice bearing SKOV-3 Xenografts, at 3 and 9 hours after intravenous injection

Organ	Uptake (% ID/g)		Tumor-to-organ ratio	
	3 h	9 h	3 h	9 h
Liver	23.1 ± 1.84	6.50 ± 1.11	0.92	0.95
Kidney	22.73 ± 2.90	6.23 ± 0.96	0.93	0.99
Spleen	13.28 ± 0.81	2.45 ± 0.69*	1.6	2.51
Blood	13.18 ± 0.42*	2.20 ± 0.66*	1.61	2.79
Stomach	4.31 ± 0.20*	0.89 ± 0.39	4.93	6.9
Lung	0.88 ± 0.04*	0.16 ± 0.06*	24.16	38.38
Tumor	21.26 ± 1.27	6.14 ± 1.45	−	−
Bone	0.48 ± 0.14*	0.07 ± 0.03**	44.29	87.71
Thyroid	0.44 ± 0.02*	0.12 ± 0.04**	48.32	51.17
Heart	0.41 ± 0.12*	0.09 ± 0.02**	51.85	68.22
Intestines	0.40 ± 0.14*	0.11 ± 0.04*	53.15	55.82
Muscle	0.23 ± 0.07*	0.04 ± 0.01**	92.44	153.5

* Significant difference of the % ID/g between tumor and other organs (*p* < 0.05).

** Significant difference of the % ID/g between tumor and other organs (*p* < 0.01). % ID/g = percent injected dose per gram of tissue.

### γ-Camera imaging

**Figure 4:**
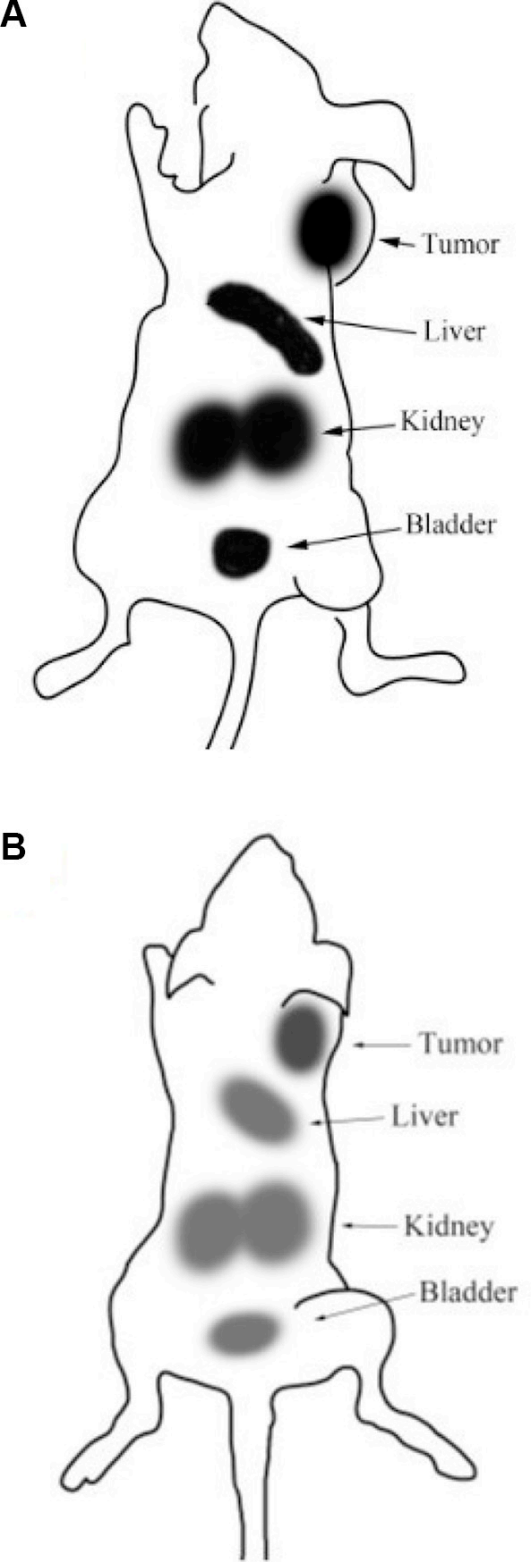
γ-scintigraphic image of the SKOV3-bearing mice after 3 hours (A) and 9 hours (B) of the i.v. injection of 99mTc-K237/FA-PEG-PLGA (LA/GA = 80/20) NPs

The scintigraphic images of the ^99m^Tc-K237/FA-PEG-PLGA (LA/GA =80/20) NPs were acquired at 3 and 9 h (Figure 4) after tail vein injection, and confirmed the results of the biodistribution experiments. These images reveal the high level of radioactivity accumulation in tumor, liver, kidney and bladder. The results of the region of interest (*ROI*), show that the T/NT of tumor and contralateral muscle conforms to the biodistribution experiments. The uptake of radioactivity by the bone marrow of the mice injected with ^99m^Tc-K237/FA-PEG-PLGA (LA/GA = 80/20) NPs was low. Additionally, compared with the radioactivity accumulation at 3 h, after 9 h of the tail vein administration of the labeled NPs, the distribution *in vivo* was the same as the 3 h and the radioactivity was decreased in the liver, kidney, bladder and tumor.

## CONCLUSIONS

K237/FA-PEG-PLGA NPs with narrow size and homogenous distribution were successfully prepared using the W/O and solvent evaporation method. The NPs loaded with drug were also prepared using a similar method [24]. These approaches can be an alternative to improve the stability and solubility of drug, while enhancing the absorption and bioavailability of the loaded-drug by tumors. The K237/FA-PEG-PLGA NPs were labeled with ^99m^Tc using a rapid and simple direct labeling procedure to achieve high labeling efficiency and stability within 24 h. Relatively high radioactivity were observed in blood, liver, kidney and tumor after i.v. administration, and high retention in blood were advantageous to increase the loaded-drug residence time. Scintigraphic images showed that the maximum uptake occurred in liver, kidney and tumor. In conclusion, the NPs could be a potential ideal drug delivery system (DDS) to improve the efficacy of the loaded-drug and reduce drug-associated toxic and side effects during cancer treatment.

## MATERIALS AND METHODS

### Materials

PEG, molecular weight (Mw) 2000, was obtained from Sigma-Aldrich Co., Ltd. (Shanghai, China). L-lactide and glycolide were purchased from Yuanshengrong Company (Beijing, China). Pluronic 188 (F68) was obtained from BASF (Ludwigshafen, Germany). Dialysis bags (MWCO 3500 Da) were purchased from Qcbio Science & Technologies Co., Ltd (Shanghai, China). N-hydroxysuccinimide (NHS) and 1,3-diisopropylcarbodiimide (DIC) were from Meloney Biotechnology Co., LTD (Dalian, China). FA was purchased from Mr. Ng Biological Technology Co., LTD (Nanjing, China). K237 peptide (HTMYYHHYQHHL) was synthesized by GL Biochem Ltd (Shanghai, China). Silica gel for thin-layer chromatography was from Qingdao Haiyang Chemical Co, Ltd (Qingdao, China). Diethylenetriaminepentaacetic acid (DTPA) and cysteine were obtained from Shanghai Yingrui Chemical Technology CO, Ltd (Shanghai, China). Cell culture media and reagents were purchased from Gibco (Grand Island, NY, USA), unless otherwise indicated. Dicyclohexylcarbodiimide (DCC), 4-dimethylaminopyridine (DMAP), SnCl_2_, ethyl acetate and other reagents and solvents were from Sinopharm Chemical Reagent Co, Ltd (Shanghai, China).

### Cells culture and animals

The SKOV-3 (ovarian cancer cell) were cultured in Dulbecco's modified Eagle's medium (DMEM) (Gibco) supplemented with 10% fetal bovine serum (FBS), 100 U/mL penicillin and 100 μg/mL streptomycin, at a 37^°^C, in a humidified atmosphere of 5% CO_2_ [25–27].

Healthy 4–6 week old male nude Balb/c mice (body weight: 20 ± 3.5 g) were obtained from the Fudan University. All experimental procedures were approved by the Animal Care and Use Committee at the Fudan University.

### Synthesis of the K237/FA-PEG-PLGA polymer

**Scheme 1:**
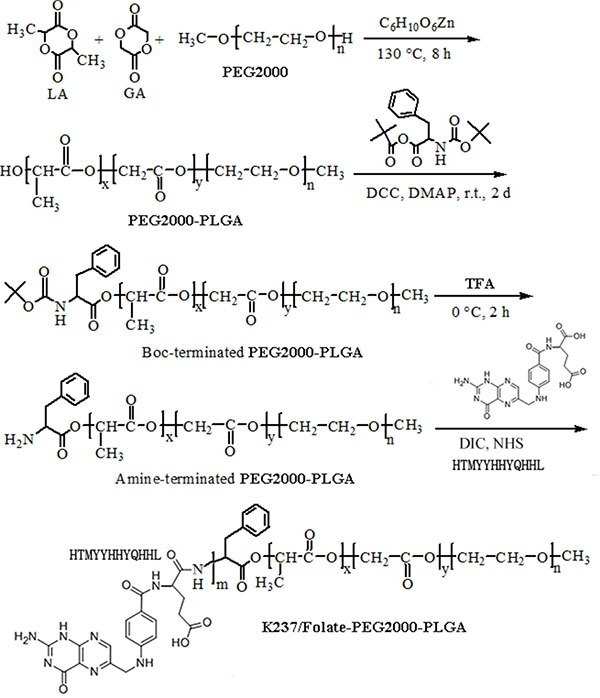
Schematic depiction of K237/FA-PEG-PLGA copolymer synthesis

The synthesis of the K237/FA-PEG-PLGA (LA/GA = 60/40, 70/30, 80/20) polymer (Mw 12,000) has been described in detail elsewhere [28–31], following the steps depicted in Scheme 1. In brief, (1) the hydroxyl-terminated PEG-PLGA was synthesized; (2) the hydroxyl end-group was then converted to Boc-_L_-Phe; (3) the t-Butoxycarbonyl end-group was removed, followed by the synthesis of the amino-terminated PEG-PLGA; (4) the amino-terminated PEG-PLGA (200 *μ*mol) was dissolved in DMSO (60 mL), then mixed with NHS (1,000 *μ*mol), DIC (1,000 *μ*mol), K237 (500 *μ*mol) and FA (500 *μ*mol) at 37^°^C. Following 24 hours incubation, the solution was mixed with 200 mL of distilled water and centrifuged at 3000 rpm. The supernatant was then collected, dialyzed and freeze-dried. The obtained product was dissolved in DMSO and the concentration of the conjugated k237/FA was determined. Various concentrations of K237/FA in DMSO were used as reference.

### Preparation of K237/FA-PEG-PLGA NPs

The K237/FA-PEG-PLGA NPs were prepared using a water in oil (W/O) emulsion method. Briefly, 4 mg of K237/FA-PEG-PLGA were dissolved in 200 μL of ethyl acetate, followed by the addition of 2.0 ml of deionized water containing 1% (W/W) of F68. Subsequently, the mixture was homogenized and emulsified by ultrasonication (400 W, 6 times × 10s) with a JY 92-II ultrasonic processor (Ningbo Scientz Biotechnology Co, Ltd, China). The resulting emulsion was rotated and evaporated under 0.5 MPa and 37^°^C. After 30 min, the emulsion was stirred gently at room temperature to evaporate the left organic solvent. Finally, the NPs were isolated by centrifugation at 15000 rpm, at 4^°^C for 30 min, and the pelleted NPs were freeze dried [32, 33].

### Physical characterization

The particle size and zeta potential (ζ) of the K237/FA-PEG-PLGA NPs were characterized using a ZetaSizer Nano ZS (Malvern Instruments Ltd., Malvern, UK). The average diameters and size distribution parameters of the NPs were obtained by dynamic light scattering. The ζ was determined under a He-Ne laser beam at a wavelength of 633.8 nm at room temperature.

The morphology of the NPs was examined with an H-800 transmission electron microscope (TEM) from Hitachi Ltd., (Tokyo, Japan). The samples were prepared by depositing 10 μL of NPs suspension on a 200 mesh, copper grid with a formvar film, and then air-drying at room temperature [34–37].

### Radiolabeling of K237/FA-PEG-PLGA NPs with ^99m^Tc

Radiolabeling of the NPs was performed by a direct method using stannous chloride as a reducing agent. The effect of the amount of NPs and stannous chloride, the final pH of the preparation and the incubation time on the labeling efficiency was previously optimized by changing a parameter at a time and by performing quality-control tests for the labeled complex as described earlier [38]. The amount of stannous chloride required for high labeling efficiency and low radio colloids was optimized in preliminary experiments, whereby it was determined that a range of 20 to 100 μg of stannous chloride was optimal and thus was used in further experiments. Similarly to achieve optimal labeling efficiency, the amount of NPs, the pH of the reaction mixture and the incubation time were also investigated. Eventually, the most appropriate labeling protocol was found and can be described as follows. Briefly, 250 μL of NPs dispersion (2 mg/mL) were mixed separately with 20 μg of stannous chloride (40 mg/mL). After adjusting the pH of the mixture to 7.0 with sodium hydrogen carbonate (0.5 M), 50 μL of a ^99m^TcO_4_^−^ solution (0.74–3.7 GBq), freshly eluted from a ^99^Mo-^99m^Tc generator (Drytec, GE Healthcare), was added to each preparation, mixed gentle, and incubated for 15 min at room temperature. Final radioactivity concentration present in the preparation was examined using a well-type gamma ray spectrometer (Cobra II Inspector 5003; Canberra Packard Central Europe GmbH, Romania) [39].

### Determination of labeling efficiency

The labeling efficiency of the NPs was determined by ascending thin layer chromatography (TLC) using instant thin-layer chromatography (ITLC) strips coated with silica gel and the test temperature was 25^°^C. The ITLC strips were used to determine the free ^99m^Tc and percentage of radio colloids in the preparation. Based on these two parameters, the labeling efficiency of the preparation was calculated [40].

ITLC strips were spotted with 2~3 μL labeled complex at 1 cm above the bottom. These strips were advanced by using acetone as the mobile phase to determine the percentage of the labeled complex and a solvent (V_pyridine_: V_acetic acid_: V_water_ = 3:5:1.5) as the mobile phase to determine free ^99m^Tc and labeled NPs. The solvent front was allowed to reach to a height of approximately 6 to 8 cm from the origin. The radioactivity in the strip of R_f_ value = 0.1 to 1.0 was determined by well-type gamma ray spectrometer. The free ^99m^Tc present in the preparation migrates to the top portion (R_f_ value about 0.8 to 1.0) of the ITLC strip, leaving the labeled NPs along with the radio colloids (reduced/hydrolyzed ^99m^Tc) at the application point when using acetone as the mobile phase. The presence of radio colloids was determined by developing the ITLC strip using a solution of pyridine: acetic acid: water in volume proportion of 3:5:1.5. The reduced/hydrolyzed ^99m^Tc present in the preparation remains at the point of application, while both the free ^99m^Tc and labeled NPs migrate to the front with the solvent front [38]. The labeling efficiency was calculated by using the following equation:

Labeling efficiency (%) = [B_acetone_/(T+B)_acetone_ – T_pyridine_/(T+B) _pyridine_] × 100%. Where, T and B are the radioactivity counts at the top and bottom of the strip, respectively.

### Stability of labeled complexes

The stability of the ^99m^Tc-labeled NPs was evaluated *in vitro* in human serum and normal saline by the ascending TLC technique. The freshly labeled complex (10 μL) was incubated with freshly collected human serum (90 μL) at 37^°^C. Following incubations for 1 h, 4 h, 9 h and 24 h, the samples were separated by ITLC using the above mentioned solvent systems.

### DTPA and cysteine challenge

The *in vitro* stability studies of ^99m^Tc-labeled NPs were performed using DTPA and cysteine as previously reported [24]. Briefly, fresh DTPA and cysteine solutions (10, 30, 50 mM, each) were prepared in 0.9% NaCl solutions. A 500 μL volume of the labeled NPs was incubated with the different concentrations of DTPA and cysteine for 1h at 37°C, while a 500 μL of 0.9% NaCl solution served as control. The effect of DTPA and cysteine on the labeling efficiency of the prepared complexes was analyzed by ITLC-silica gel strips using acetone as mobile phase. In this acetone system, the ^99m^Tc-labeled NPs remain at the point of application (R_f_ = 0.0), while free pertechnetate (R_f_ = 0.8–1.0) and all known chemical forms of ^99m^Tc-DTPA and ^99m^Tc-cysteine complexes migrate upward (R_f_ = 0.7–1.0). After developing, each paper was cut into two halves, the top and bottom halves, and radioactivity in each half was measured using a gamma-ray spectrometer [41, 42].

### Cell binding and blocking experiments *in vitro*

The specificity of the ^99m^Tc-labeled NPs for receptor was assessed *in vitro*. SKOV-3 cells in the logarithmic phase of proliferation, 80–90% confluency, were detached with trypsin and resuspended at a cell concentration of 5 × 10^6^, 1 × 10^6^, 5 × 10^5^, 1 × 10^5^, 5 × 10^4^, 1 × 10^4^ cells/mL. A volume of 1 mL of cell suspension was transferred into a centrifuge tube, then 1.5 μg (0.37-2.22 MBq, 100 μL) of ^99m^Tc-K237/FA-PEG-PLGA NPs in DMEM solution was added to each tube, followed by incubation for 1 h at 37^°^C, before the radioactivity count (T) was measured. Cells were collected by centrifugation (3000 rpm/min) for 10 min, at 4^°^C, washed twice with PBS (0.01 mol/L, pH = 7.4), and then the radioactivity of the combination with SKOV-3 cells (B) was measured. Meanwhile, non-specific binding assay was performed as above described. A total of 191 μg of FA (equivalent to 1000 times, compared with FA in 1.5 μg nanoparticles) were added to the different cell samples, followed by the addition of the same amount of ^99m^Tc-K237/FA-PEG-PLGA NPs after incubation for 45 min at 37^°^C. After 1 h, the samples were centrifuged (3000 rpm/min) for 10 min, cells were collected and wash twice with PBS (0.01 mol/L, pH = 7.4), and then the radioactivity was measured. The specific cells binding rate (%) = the mean of total cells binding rate (B/T) −the mean of non-specific cells binding rate [43–46]. The cells binding rate of the ^99m^TcO_4_^−^ was determined as follows: a 100 μL (0.37–2.22 MBq) of ^99m^TcO_4_^−^ solution was added to the cells suspension solution, then the other steps were as described above.

### *In vivo* studies

#### Biodistribution studies

Previous studies have revealed that K237/FA-PEG-PLGA (LA/GA = 80/20) has better physicochemical properties and biocompatibility [27–30, 47–49]. Accordingly, the tumor-targeting properties of the preparations with the most favorable biodistribution, K237/FA-PEG-PLGA (LA/GA = 80/20) NPs was studied in mice xenografted with SKOV-3 cells. For inoculation, SKOV-3 cells (1 × 10^6^) were implanted in the right hind leg of immunodeficient mice [50–52]. Additionally, 3.7 MBq of ^99m^Tc-labeled K237/FA-PEG-PLGA (LA/GA = 80/20) NPs in 150 μL of normal saline were administered into the mice tail vein. At 3 h and 9 h post-injection mice were sacrificed by cervical dislocation and tissue were dissected for measurement of biodistribution and tumor targeting. The radioactivity associated with the blood, heart, liver, kidney, tumor, lung, spleen, stomach, muscle, and bone tissues were examined with a gamma counter, together with a standard radioactive solution of known quantity administered at the time of each injection, which was considered as 100%. The radioactivity present in each tissue sample was expressed as percent injected dose per gram of tissue (% ID/g) (*n* = 6) [53, 54].

### γ-Camera imaging

*In vivo* imaging was performed to obtain a visual confirmation of the biodistribution data. The SKOV-3 xenograft-bearing mice were injected with 100 μCi of ^99m^Tc-K237/FA-PEG-PLGA (LA/GA = 80/20) NPs. Before imaging, mice were anesthetized with a mixture of 18.75 mg/kg ketamine hydrochloride and 0.5 mg/kg medetomidine hydrochloride. The accumulation of radioactivity in mice was monitored by imaging with a γ-camera (GE Healthcare, Cleveland, OH, USA) equipped with a low-energy high-resolution collimator. Static images were obtained by a 512×512 matrix and a pixel side length of 0.4 mm, resulting in a total imaging time of 757 seconds. [55–58]

### Statistics

Statistical analysis was performed using the SPSS 20.0 software (IBM Corp., Armonk, NY, USA). All results are expressed as mean ± standard deviation (SD) for the values obtained from a minimum of three independent experiments. All statistical analyses involving comparisons of multiple groups were performed using the one-way analysis of variance (ANOVA) and Tukey's post hoc test. All tests were considered statistically significant when the *P* value was less than 0.05.
